# A challenge for healthcare but just another opportunity for illegitimate online sellers: Dubious market of shortage oncology drugs

**DOI:** 10.1371/journal.pone.0203185

**Published:** 2018-08-28

**Authors:** András Fittler, Róbert György Vida, Valter Rádics, Lajos Botz

**Affiliations:** Department of Pharmaceutics and Central Clinical Pharmacy, Faculty of Pharmacy, University of Pécs, Pécs, Hungary; Brighton & Sussex Medical School, Falmer, UNITED KINGDOM

## Abstract

**Introduction:**

Drug shortages mean a challenge to healthcare systems. Exposed patients or health care providers may seek alternative resources for these products online. The purpose of our study was to analyze the online availability of oncology shortage drugs at national and at international levels in 2014 and 2016.

**Methods:**

We tested the online accessibility of oncology shortage drugs by simulating the Internet search method of patients. Search results were evaluated according to operational, distributional, and patient safety characteristics.

**Results:**

In 2014 and 2016 all (100%) antineoplastic agents affected by shortages were available on the Internet without medical prescription. The number of relevant websites among search engine results has decreased from 112 to 98, while online vendors actually offering oncology shortage drugs for sale has risen from 66.1% to 80.6% within relevant websites in the two evaluated years. None of the online sellers were classified as legitimate or accredited by LegitScript and VIPPS online pharmacy verification databases.

**Conclusion:**

According to our findings shortage oncology drugs are widely available online. To manage shortages and illegal Internet trade national and international standardized shortage reporting and information systems, regularly updated Internet pharmacy verification databases are needed. As well, institutional procurement and medication use review policies are required.

## Introduction

### Drug shortages

Drug shortages induce a complex global public health crisis [1–5] and have a great impact on provision of healthcare services from the early 2000s [6] as they are posing a challenge to ensure medication and patient safety [7]. Although there are different approaches in defining drug shortages all definitions emphasize a critical drug supply issue requiring change and affecting patient care [2, 5, 8–10].

Although health systems all over the World are troubled by with the short supply of medicines, the literature still lacks robust data. In the United States the number of annual new shortage was the highest in 2011 with 257 entities and decreased to 136 by 2015. However, the total number of shortages (new and ongoing) in these years was nearly the same (441 and 427). Alternatively, in Europe and Australia only surveys are available [11].

European hospital pharmacy surveys (Pauwels 2013, EAHP 2014) showed that lifesaving medications (oncology, emergency, infectology) were regularly affected by shortages and almost every hospital was facing the problem in the 2013–2014 years, similarly to the United States [1, 12]. Also, it is well known that shortages unevenly impact sterile, generic injectable medications and are less likely to affect branded oral tablets [12, 13].

The main difference between the U.S. and European drug supply issues is that the generic/originator ratio of drugs being in shortage is much higher in the United States comparing to the European countries [11, 14].Causes and contributing factors of drug shortages may vary in countries and show a multidimensional nature both on the supply and demand side. Despite the variation there are similarities in the primary drivers, such as manufacturing problems, economic and health policy decisions [2, 15, 16–22] In Europe the leading cause of drug shortages can be linked to manufacturing and GMP compliance problems [23].

Shortages can result in compromised quality, safety and efficacy in the supply chain, with numerous negative therapeutic and financial consequences on patient care. In the United States fifteen deaths happened due to shortages between 2010 and 2011 [2, 24, 25–27].

Managing drug supply problems can lead to additional expenses (purchasing cost, labor cost, and cost of under-treatment) and higher workloads as well. The latter may decrease direct patient care activities which can lead to an increasing number of medication errors [2, 3, 24, 25, 28, 29]. Furthermore, other consequences not necessarily involved in the traditional supply chain also exist, such as the acquisition of unapproved or falsified medication from alternative sources [30–32].

Injectable oncology drug shortages are widespread, recurrent and persistent with a significant impact on patient care [16, 33–39]. In the United States anti-infective and oncology drugs are the most affected therapeutic areas in the past five years. In Europe, antimicrobials, oncology, and emergency medicines are most commonly in shortage, showing similarities with other parts of the world according to the survey of the EAHP [40–43].

However, we do not have exact data on the extent of the problem. According to recent estimates, annually over 500,000 oncologic patients are affected by drug shortages in the United States [44, 45]. In Europe the 2014 EAHP survey found that 55% of more than 500 hospital pharmacist respondents from 36 countries have experienced oncology drug shortage in 2013 [12].

### Illegal Internet trade of pharmaceuticals

Despite a growing number of legitimate Internet pharmacies are offering their services, a vast number of illegitimate vendors overwhelm the market of online pharmaceuticals [46–50]. The patient safety risk attached to the online purchasing of shortage drugs involves questionable sourcing, poor quality, falsification, improper storage and transportation [50–52]. Further, there are not only medical but also financial consequences associated with procuring medications from the web as the price of online drugs is documented to be much higher than offered by traditional brick and mortar pharmacies [53–55].

It has been proven in the literature that countless medications can be ordered online and uninformed consumers are unlikely to be able to differentiate legitimate websites from illegitimate actors [40, 56]. Unregulated online pharmacies are willing to supply prescription only drugs without valid medical prescriptions, use falsified verification logos and conceal their contact details [32, 40, 46, 57]. In addition, professional aspects of safe medication use are deteriorated by unethical marketing practices [55], absence of product information or valid live contact with healthcare professionals (pharmacist or physician) [53, 56, 58].

Previous studies showed that drugs in shortage (vaccines and oncology drugs) are available on the Internet from NABP not recommended vendors, a detailed and follow up analysis of the availability of shortage oncology drugs via the Internet has not been carried out so far [52, 59].

## Objectives

The objective of our current study was to analyze the online availability of oncology medications affected by national and international drug shortages in 2014 and 2016. By the evaluation of Internet search results and vendor websites the illegitimate accessibility of such specific and explicitly life threatening medications can be highlighted. Online availability of shortage drugs—in general—has been documented earlier by Liang BA [47], however mainly focusing on Food and Drug Administration (FDA) shortage-listed drugs and not evaluating European and international shortage issues and the survivability of Internet pharmacy websites.

## Materials and methods

We tested how easily patients could access out-of-stock oncology drugs via the Internet by simulating the Internet search method of patients. Search engine results, all relevant links, web pages and drug vendor sites (Internet pharmacies) were assessed in a descriptive study performed in 2014 and 2016. Researchers assessed websites anonymously and did not complete any purchases during the study, therefore no ethical approval was considered necessary.

### Identification of national and international drug shortages

The 2014 EAHP survey on drug shortages was the starting point to identify drug shortage information sources. We accessed these publicly available shortage databases. Inclusion criteria were: relevant shortage information on oncology medications and date of shortage events (2014 and 2016) of shortage event. Publicly available databases with Hungarian or English language were preferred [12]. See Table A in S1 Table.

Seven European and 4 Non-European drug shortage information sources (list, database) were included.

The query of shortage antineoplastic agents in the databases was performed by ATC code (L01) or the name of the Active Pharmaceutical Ingredient (API) from July 01. to September 30. (Q3) in 2014 and 2016. If a database only contained brand name of a drug product then the national authority’s pharmaceutical product database was searched to determine the API or the therapeutic class (ATC code). ATC L01 Active Pharmaceutical Ingredients were identified according to the WHO Collaborating Centre for Drug Statistics Methodology website (https://www.whocc.no/).

An active ingredient was considered to be an *internationally significant shortage problem* if it was represented in at least one accessible European database (aside from the Hungarian) and in addition in at least one Non-European shortage database [12, 60–70]. See Table B in S1 Table

### Obtaining search engine results

We searched Google (www.google.hu) for the English and Hungarian terms of antineoplastic agents (ATC L01 class drugs) affected by shortages in July-September (the third quarter of the calendar year, Q3) both in 2014 and 2016. The name of the active pharmaceutical ingredient (API—according to the WHO terminology) and the term “buy” were used as specific search key words (e.g.: “buy bleomycin”). Web browser Firefox was used with standard security setting, with no user signed in any account during searches. By documenting the first 50 references appearing in Google for national (Hungarian) and English language search terms, we could simulate what patients can easily find and what websites they most likely would visit when searching for shortage oncology drugs. No Hungarian websites were identified, consequently only websites operating in English were included in our study. No direct searches were performed on social media sites such as Facebook, Twitter, etc..

A search engine result was classified as relevant if the title and/or short description (so called title- and meta-description tags) indicated possible purchase option for the given active pharmaceutical ingredient. Accessed websites were categorized as *Internet pharmacies* (direct online sellers), *intermediary sites* (websites that were not direct vendors but acted as sources of information to purchase drugs), *social media sites* (blogs and forums); *research only sites* (companies offering active ingredients for research), and the remaining as *others* (e.g.: wholesalers) similarly to previously published studies [40, 47, 53]. Only Internet pharmacies were included in our study for detailed evaluation, while vendors offering non-finished products, raw materials or chemicals for scientific use were excluded.

### Evaluation of Internet pharmacy websites

The identified direct online drug sellers were assessed according to the following characteristics: *Operational characteristics* (website category, year of accessibility, oncology specific domain name: a domain name containing expressions related to oncology e.g.: *cancer*curepharmacy.com, contact information: address/location, telephone number), *Distributional characteristics* (drug availability, price, quantity limit), and *Patient safety characteristics* (requirement for a medical prescription, possibility of information exchange with healthcare professionals, LegitScript [71] and VIPPS [72–74] legitimacy certifications). Data was documented according to previously defined methodology by the authors. Websites were identified by domain name. The domain name was used during survivability and legitimacy evaluations. The authors assessed the possibility of purchasing products by placing an order for an oncology shortage medication and aiming to proceed to the checkout page, but no product purchase was made due to financial, ethical and safety concerns.

### Statistical analysis

The data was summarized by descriptive analysis. Mann-Whitney test and Chi-Square test were used to evaluate correlations.

## Results

### Online access to shortage oncology drugs

**Fig 1:**
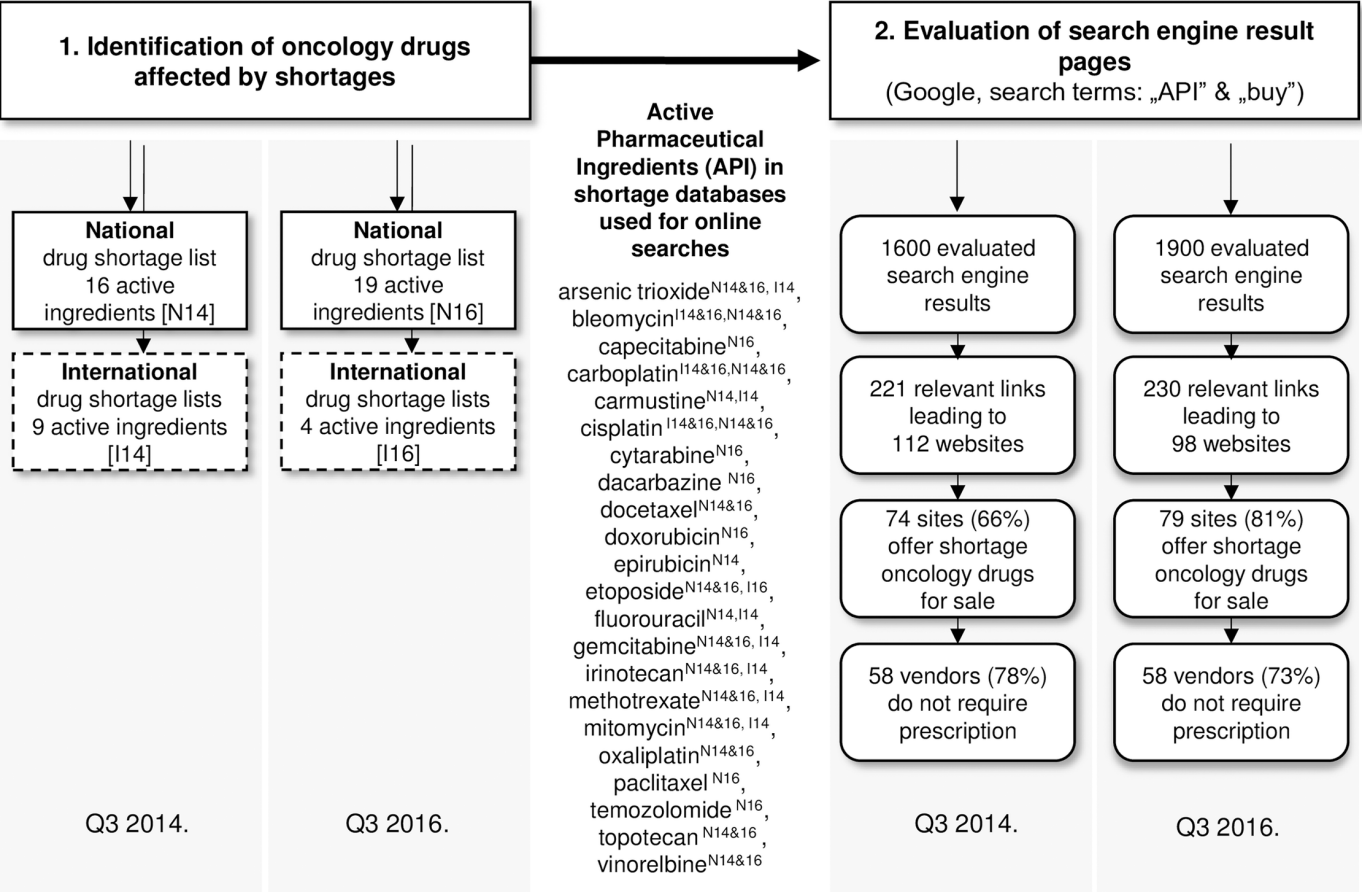
Flowchart illustrating methodology and main results regarding online availability of and prescription requirement for shortage oncology active pharmaceutical ingredients. Letters indicate geographical localization of evaluated databases N: national, I: international; numbers indicate year of evaluation 14 (2014), 16 (2016); third quarter of the calendar year is labelled as Q3.

All evaluated oncology drugs affected by shortages were available on the Internet as each API included in our study (100%) was offered by online pharmacies both in 2014 and 2016. As can be seen from **Fig 1.**, numerous search engine results (13.8% and 12.1% in 2014 and 2016) contained relevant information about purchasing shortage oncology medications. Of these relevant links 221 led to 112 individual websites in 2014, while 230 to 98 websites in 2016. The average number of websites per API somewhat decreased from 7 (2014) to 5.15 (2016). The ratio of online pharmacy sites actually offering finished products for sale within all relevant websites has risen from 66.1% (2014) to 80.6% (2016) (p<0,001).

About every fourth online vendor (n = 14) was listed within the first fifty search engine results in both years of our study. Consequently, these sites were active, relatively easy to access and continuously available for patients in the past two years. In one case, a different domain extension was used for the same domain name (e.g.: buy-pharma.com & buy-pharma.co), indicating multiple (bulk) domain name operation. The accessibility of all Internet pharmacy websites (n = 74) identified in 2014 was evaluated two years later and one fourth (n = 19. 25.5%) were functioning in both years which is a clear sign of continuous operation. An online pharmacy was considered to be an active/functional vendor if payment options were displayed on the website (e.g. credit card information was requested) as the final step of purchase during the checkout procedure. Sixty-four (86.5%) Internet pharmacies in 2014, while seventy-seven (97.5%) in 2016 vendors were active/functional.

Online vendors generally did not require medical prescriptions as 78.4% (58/74) internet pharmacies in 2014 and 73.4% (57/79) in 2016 offered oncology medications without the necessity of such medical documentation (p = 0.474). One Internet pharmacy found in 2016 emphasized “no prescription required” access on its website, while of the websites used this marketing message in 2014. Eight (10.8%) Internet pharmacy websites have used oncology specific domain names containing the terms e.g. “cancer” or “oncology” in 2014, down to five (6.3%) in 2016 (p = 0.320).

The shortage oncology portfolio of websites was similar in both evaluated years. The maximum number of shortage drugs offered per website has somewhat increased from 12 to 13, while the average number of shortage oncology medications offered per website was similar: 2.12 (±2.293) and 2.15 (±2.602) in 2014 and 2016 respectively (mean±standard deviation; Man-Whitney U test p = 0.784). Most of the evaluated active ingredients became available on a larger number of Internet pharmacies. A notable decrease was observed for others: number of carboplatin (p<0.05) and methotrexate (p<0,001) products offered has significantly decreased by 2016.

In accordance with previous studies we have also documented that numerous vendors have not displayed any physical address or telephone number on their websites (20.3% in 2014 and 27.8% in 2016). Furthermore, no healthcare professionals–pharmacist or medical doctor–were accessible on most Internet pharmacies (90.5% in 2014 and 91.1% in 2016). The majority (86.5%) of the identified vendors in 2014 and all (100%) in 2016 offered shipment to European countries. There was generally no limitation by the majority (91.9% in 2014 and 97.45% in 2016) of the Internet pharmacies on the amount of medication that could be ordered.

### Verification according to LegitScript and VIPPS

LegitScript is a private company monitoring Internet pharmacies, supplement sellers and other online merchants. The company maintains a uniquely large database of more than eighty thousand Internet pharmacies. To evaluate whether online pharmacy websites included in our study were definitely fraudulent, we checked each domain name within the LegitScript database in Q3 2014 and Q3 2016. Internet pharmacy websites are classified by LegitScript into four categories: legitimate, unverified, unapproved, and rogue. “Legitimate” Internet pharmacies have passed LegitScript certification criteria and meet the standards of the National Association of Boards of Pharmacy (a US independent and international association that assists its member boards for the purpose of protecting the public health). “Unverified” websites appear eligible for certification, but have not been subjected to LegitScript’s certification or voluntary monitoring program. “Unapproved” sites have problems with regulatory compliance or risk and do not comply with LegitScript’s verification standards or applicable laws or regulations but do not meet the definition of being rogue. “Rogue” Internet pharmacies are illegitimate websites. Their primary purpose is to engage in some sort of illegal, unsafe, or misleading activity, like selling prescription drugs without requiring a prescription, defrauding customers, or selling counterfeit goods [40, 45, 71]. Websites that are not evaluated by the organization are labeled as “Not available in the database”.

In the United States, the NABP operates VIPPS, a voluntary accreditation program. “Accredited” websites address a customer’s right to privacy, authentication and security of prescription orders, adherence to a recognized quality assurance policy, and provision of meaningful consultation between customers and pharmacists. “Not recommended” vendors appear to be out of compliance with state and federal laws or NABP patient safety and pharmacy practice standards [40, 72, 73, 74].

Website legitimacy is most often evaluated by the LegitScript and the VIPPS Internet pharmacy verification databases in relevant literature. In our samples obtained during the two evaluated years none (0%) of the identified Internet pharmacies were classified as “Legitimate” or “Accredited” by these verification databases, and a notable number of definitely unregulated websites offered products amongst the search engine results. There is a significant difference in the categorization of websites in case of the databases between the two evaluated years (see **Table 1**). However, a realignment can be observed between the definitely unregulated participants and ones that are not available in the databases. Seemingly the ratio of illegal actors somewhat increased and the completeness of databases has simultaneously slightly improved. The legitimacy of online pharmacies was assessed in 2014 Q3 and 2016 Q3 (July-September) within 2 weeks after the identification of the online pharmacies in google searches.

**Table 1:** Website legitimacy according to LegitScript and VIPPS Internet pharmacy verification databases.

Database	Internet pharmacy category	Time of evaluation	
		Q3 2014 n (%)	Q3 2016 n (%)
LegitScript	Rogue	23 (31.1%)	48 (60.8%)
	Unapproved	18 (24.3%)	11 (13.9%)
	Unverified	0 (0%)	0 (0%)
	Legitimate	0 (0%)	0 (0%)
	Not in database	33 (44.6%)	20 (25.3%)
VIPPS	Not recommended	11 (14.9%)	26 (32.9%)
	Recommended	0 (0%)	0 (0%)
	Not in database	63 (85.1%)	53 (67.1%)

Chi-square test: p = 0.001 for LegitScript; p = 0.009 for VIPPS. Note: It is *likely that not only the number of illegal websites have increased, but that the databases have evolved during the time of our study. For example the LegitScript database contained approximately 35 000 websites in 2014, while nearly 81 000 in 2016 [71, 72].*

We must mention that LegitScript website only allows the limited number of five pharmacy verifications per day, which seems to be a disadvantage from the aspect of patients seeking safe online sellers. Also, an important limitation of the VIPPS website was noticed. The online tool was not able to identify explicit illegal websites that are otherwise listed among “Not Recommended Sites” by the NABP itself [71, 72]. The EU common logo for legally operating online pharmacies/retailers in the EU Member States was introduced by the Falsified Medicines Directive (2011/62/EU) and operates since the 1st of July 2015. This logo is another opportunity to recognize website legitimacy. However, the authors did not document the presence of this seal as it was not obligatory when the study was initiated in 2014 [75].

### Comparison of the national and international shortage situation

The authors aimed to evaluate if their findings based on national shortage conditions could be generalized and if the results could characterize a global phenomenon. Internationally significant active ingredients were identified based on methods described earlier and key findings of the study were correlated with results based on the national drug shortage lists. With the assessment of the Non-European shortage lists, and the seven accessible European national shortage databases, we have identified nine oncologic drugs that were in shortage globally in 2014 (bleomycin, carboplatin, carmustine, cisplatin, fluorouracil, gemcitabine, irinotecan, methotrexate, mitomycin) and four (bleomycin, carboplatin, cisplatin, etoposide) in 2016 (see also Fig 1.). See Table B in S1 Table

As it can be seen in **Table 2,** the main evaluated parameters show similarity for national and international shortage products. Therefore the key findings of our study are supported by data obtained based on internationally significant drug shortages.

**Table 2:** Comparison of key findings of national shortage list APIs and internationally significant shortages.

Evaluated parameters and online accessibility findings	2014 shortages		2016 shortages	
	16 national	9 internationally significant	19 national	4 internationally significant
Relevant links and websites within first 50 search engine results	221 links of 112 websites	134 links of 72 websites	230 links of 98 websites	53 links of 33 websites
Internet pharmacies within individual relevant websites	66.07% (74/112)	76.4% (55/72)	80.6% (79/98)	66.6% (22/33)
Number of shortage oncology drugs identified per pharmacy (mean±standard deviation)	2.12 ±2.29	2.33 ±2.57	2.15 ±2.602	4.36 ±4.11
Online pharmacies not requiring prescriptions for oncology drugs	78.4% (58/74)	78.2% (43/55)	73.4% (58/79)^1^	77.3% (17/22)
Oncology specific domain name	10.8% (8/74)	12.7% (7/55)	6.3% (5/79)	9.1% (2/22)
Legitimacy according to LegitScript				
Rogue	23 (31.1%)	16 (29.1,0%)	48 (60.8%)	15 (68.2%)
Unapproved/Unverified	18 (24.3%)	11 (20.0%)	11 (13.9%)	3 (13.6%)
Legitimate	0 (0%)	0 (0%)	0 (0%)	0 (0%)
Not in database	33 (44.6%)	28 (50.9%)	20 (25.3%)	4 (18.2%)
Legitimacy according to VIPPS				
Not recommended	11 (14.9%)	9 (16.4%)	26 (32.9%)	9 (40.9%)
Recommended	0 (0%)	0 (0%)	0 (0%)	0 (0%)
Not in database	63 (85,1%)	46 (83,6%)	53 (67.1%)	13 (59.1%)

## Discussion

### Drug shortage information sources and databases

Due to this relatively novel and serious global pharmaceutical market issue publicly available drug shortage information is needed to handle and manage product supply disruptions and their consequences [5, 76, 77]. National online shortage catalogues can provide valuable information for healthcare personnel and policy makers on the extent and nature of the problem at a country level [1]. Additionally, available shortage information may also prevent patients from “gray market” suppliers and buying counterfeit and illicit medications through the Internet [77].

In 2011, President Obama issued an Executive Order emphasizing the importance of notification in helping Food And Drug Administration (FDA) to prevent drug shortages [78], while international recommendations by the International Pharmaceutical Federation (FIP) and the European Association of Hospital Pharmacists (EAHP) also highlighted the establishment of publicly accessible information resources on medicine shortages [2, 12]. By this time almost every health authority and medicine agency has taken steps to collect information proactively from the manufacturers and to provide shortage information on their websites. Extensive databases are available in the United States, Canada and Australia [1, 70, 79]. According to the 2014 survey of the EAHP shortage information or catalogues are also available in most European countries as well [2]. Although there is a centralized database collecting information about drug shortages in Europe that is maintained by the European Medicines Agency (EMA), unfortunately it only contains data that has been assessed by the Agency [1, 23, 66].

**Fig 2:**
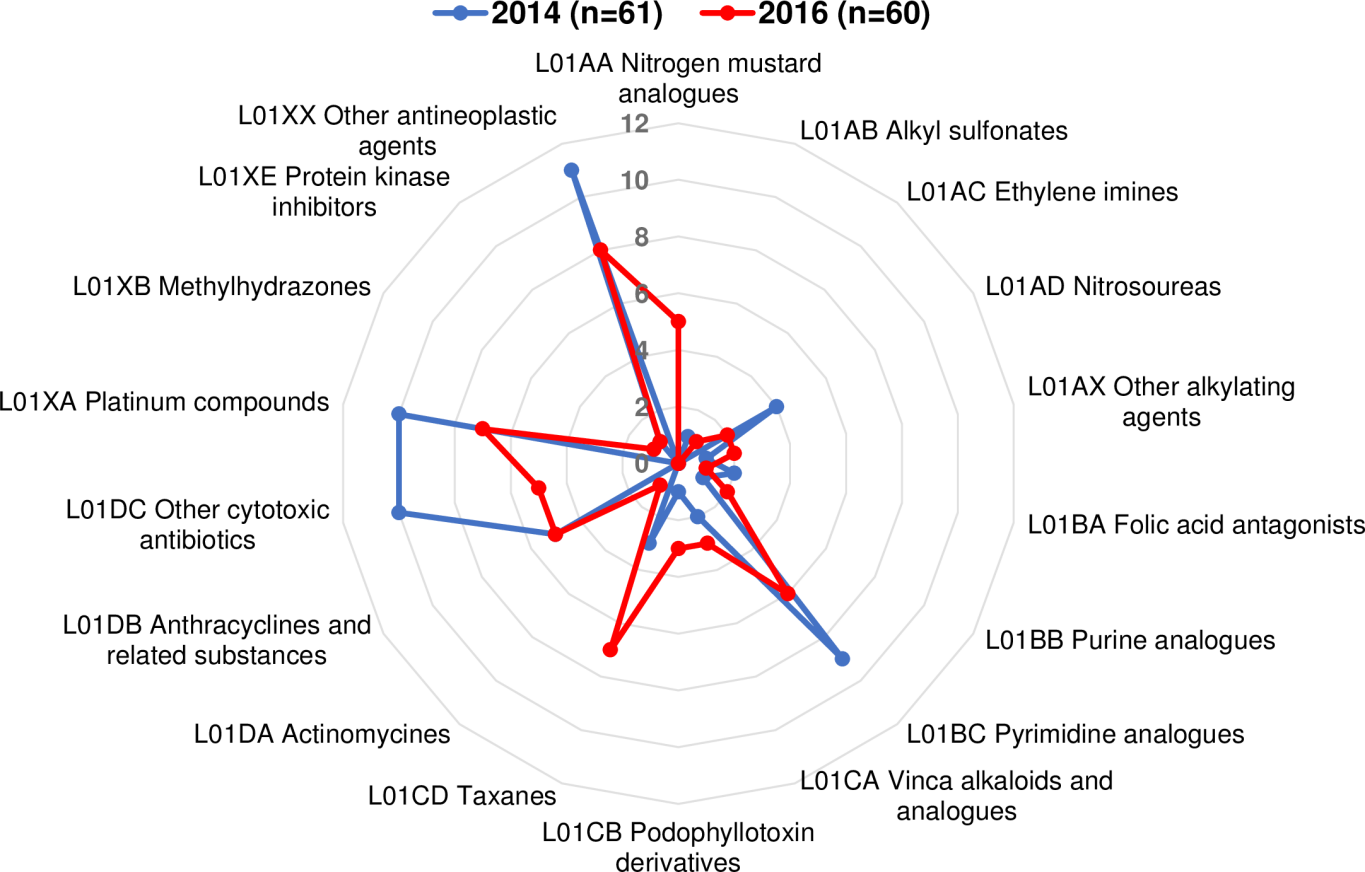
The pattern of identified oncology shortage active ingredients in 2014 and 2016.

Following the analysis of eleven drug shortage databases, thirty seven active pharmaceutical ingredients (20% of 178 L01 ATC) were identified by the authors in 2014 and 2016 (Table B and C in S1 Table). We wanted to highlight the time pattern of oncology drug shortages during the two evaluated years. As it can be seen in **Fig 2,** the total number of documented shortage events (active pharmaceutical ingredient on a shortage list) are nearly the same in 2014 (n = 61) and 2016 (n = 60) and represent a wide range of fourth level chemical ATC subgroups [80]. However, it is important to note that the shortage status and pattern of active ingredients changed over time. We believe that systematic data collection and the analysis of shortage patterns over time supports better understanding of shortage issues.

### Drug shortages and the Internet

Drug shortages turned into a global healthcare problem affecting safe and cost-effective patient care in most countries. Managing medication shortages requires a multi-disciplinary approach that integrates medical doctors, pharmacists and policy makers.

In order to combat product supply disruptions, regularly updated and publicly available drug shortage information is essential. Oncology is one of the most commonly affected therapeutic areas concerning shortages in most developed countries [36]. The limited access to pharmaceuticals is most likely a strong motivator for patients and healthcare professionals to obtain treatments outside the traditional drug-supply-chain.

Internet trade is among the most convenient alternative purchasing options for medications. Unfortunately, the online medication market is currently dominated by illegitimate vendors offering products without medical prescriptions and adequate professional supervision [40, 46, 81]. The chance of obtaining illegitimate, substandard or counterfeit dugs from the Internet is high as currently there is no effective international supervision and law enforcement of the online pharmaceutical market. For drugs in short supply, providers and hospitals search beyond conventional sources to obtain the medications. Thus, shortages of key medicines have created opportunities for illegitimate traders for price markups, while also increasing the chance of infiltration of counterfeits to the market [82, 83].

### Online accessibility of oncology drugs

The authors aimed to evaluate the online accessibility of oncology drugs affected by drug shortages to highlight the dangers of the unregulated sale of possibly life threatening products. Oncology drugs affected by shortages were identified by evaluating national and international drug shortage databases, while online availability was evaluated by searching for these shortage antineoplastic agents with the popular search engine Google. Direct online drug sellers were assessed for operational, distributional and patient safety characteristics.

Oncology drugs affected by shortages were generally available on the Internet. Accordingly, it seems that these vendors do not differentiate between plentiful and shortage drugs, and can offer both categories for sale. Numerous Internet vendor sites (25.5%) operated in both evaluated years, indicating illegitimate sellers’ continuous service and broad product portfolio. Current findings are in line with our previously published results on long-term follow-up of Internet pharmacies [40].

Shortage oncology medications were accessible without medical prescription by the majority (73.4–78.4%) of websites. Similarly to previously published studies on Internet pharmacies the authors have documented that numerous (20.3% in 2014 and 27.8% in 2016) vendors have not displayed any contact information on their websites and in most cases (90.5% and 91.1% in 2014 and 2016 respectively) no healthcare professional was available for customers’ consultation. None of the evaluated websites were legitimate (approved or recommended) by the VIPPS or the LegitScript Internet pharmacy verification databases. Nearly one third of the websites (31.1%) were definitely unregulated (rogue)—thus explicitly dangerous—by the LegitScript database in the year 2014 and seemingly the ratio of illegal actors increased (60.8%) within two years. However, simultaneously the completeness of databases has improved.

The analysis of shortage catalogues showed that the drug shortages identified in Hungary are also represented internationally (Europe, North-America, Australia), although with somewhat different appearance and duration. However, the identified number of the internationally significant shortages can be considered low (9 and 4). The reason for this may originate from the lack of completely developed data collection and database structures in the field of shortage reporting. If we look at the result of surveys and the scientific literature, there is a greater number of oncology shortage drugs in Europe and United States as well [38, 39, 77, 84]. The authors believe that their findings illustrate a global phenomenon as the comparison of key findings of our study based on national shortage list were comparable with the search results based on internationally significant shortages.

It is highly likely that anyone searching online for drugs in shortage will encounter a suspect seller, even when only searching for product information rather than with the primary intention of buying a product. It should be emphasized and communicated to patients, healthcare professionals and policy makers that not only shortages but also the online sales of oncology drugs that are in short supply pose a global problem as shown in our study. It represents a high risk to the patient care as these products are not available through the closed drug supply chain, but are obtainable over the Internet.

A gray market of shortage oncology drugs has been identified by the current study. It seems that the limited access to pharmaceuticals within the traditional supply-chain is a challenge for healthcare while being just another opportunity for illegitimate online sellers.

A multidisciplinary approach—including law enforcement, regulatory agencies, manufacturers, and customs—is required to combat both drug shortage issues and the illegal Internet trade of medications. The problems and challenges can only be resolved with harmonized actions at international, national and local institutional levels [25, 40, 85–89].

### Strengths and limitations of the study

The main strengths of our study include: up-to-date information regarding the joint threat of oncologic drug shortages and illegal Internet pharmacies, deeper insight into the temporality and relationship of these two threats and their possible effects on healthcare systems compared to previous studies [*47, 52, 59*]. A representative number of eleven drug shortage databases and internationally significant drug shortages were identified and their Internet availability was assessed. Also an inadequate function of the NABP online verification page was discovered.

This study also has limitations which need to be pointed out. The main limitation is that the complete risk based safety mapping [53] of online pharmaceutical market was not carried out, as the medications were not purchased. Actual purchases of oncology products would have provided a more in depth analysis and could have pointed out further patient and medication safety risks. Evaluation of the transportation, storage, packaging, product and patient information leaflet contents, and the analysis of chemical and microbiological quality would have added further useful data. It should be noted that test purchase of oncology drugs raises several legal, ethical and safety issues. Among numerous factors, unfortunately geographic location and language of search terms can affect search engine results. The Authors aimed to perform standardized searches and exclude different sources of bias as described in Methods. Regional differences originating from geographic location and language can be minimized if English terms are used during searches and first five search engine result pages (fifty results) are all evaluated. Accordingly, regardless of geographical location, the most visited online pharmacy websites can be identified representing European setting.

The authors had to face a methodological limitation as the quality of shortage information presented in the databases is highly variable and unfortunately difficult to assess [90]. However, the authors think that currently the best method to describe this global phenomenon and tendencies is based on the assessment shortage databases.

## Conclusion

Healthcare systems, institutions and patients are facing new challenges with the increasing number of shortage drugs [82, 84, 85, 91, 92–98] and the possibilities of purchasing medicines outside the traditional drug-supply-chain [46]. Currently international legislation and national authorities cannot guarantee continuous drug supply in several therapeutic areas affected by shortages and also the effective measures assuring safe internet sale of pharmaceuticals are still lacking. However, these problems are strongly related as the unavailability of medications, especially in the case of life saving therapies, can be a driving factor to procure pharmaceuticals from online vendors [82, 86]. Health institutions, clinicians and pharmacists may not be aware that patients can buy drugs from uncertain and illegitimate sources and these products are likely to influence the success of therapies [51]. The most vulnerable group is the patients as they are unable to weigh the potential benefits and actual dangers of using medicines from illegitimate sources. To avoid the penetration of illegal, substandard and falsified products a holistic approach is needed in combining international and national preventive measures, local institutional policies and public awareness campaigns [56].

## Supporting information

S1 Table Drug shortage databases and shortage drugs (Table A: Drug shortage databases 1.; Table B Drug shortage databases 2.; Table C: Shortage L01 ATC 3. and 4.).
(XLSX)

S2 Table Online pharmacies. (XLSX)
